# A retrospective autopsy study of 42 cases of stillbirth in Avicenna Research Institute

**DOI:** 10.1186/s12884-022-04822-9

**Published:** 2022-06-23

**Authors:** Haleh Soltanghoraee, Maziar Moradi-Lakeh, Narjes Khalili, Azadeh Soltani

**Affiliations:** 1 Reproductive Biotechnology Research Center, Avicenna Research Institute, ACECR, Tehran, Iran; 2 Preventive Medicine and Public Health Research Center, Psychosocial Health Research InstituteDepartment of Community and Family Medicine, School of Medicine, Iran University of Medical Sciences, Tehran, Iran

**Keywords:** Stillbirth, Autopsy, Causes of mortality

## Abstract

**Background:**

According to the World Health Organization about 2.6 million deaths were reported worldwide in 2015. More than 98% of stillbirths occur in developing countries. At present, the causes of many cases of stillbirth are unknown due to the lack of necessary data and autopsies in Iran. The aim of this study was to investigate the most plausible cause of stillbirth by evaluating clinical records and autopsies.

**Methods:**

A cross-sectional study of 42 stillbirth autopsies in Avicenna Research Institute from 2012 to 2019, was conducted. Data were extracted from a checklist prepared by the project researchers. The checklist contains maternal demographic information, medical history and maternal illness, pregnancy risk factors, placenta and stillbirth information. Collected data were reviewed and classified according to the ReCoDe (Relevant Condition at Death) system.

**Results:**

In the present study, based on ReCoDe classification, related causes of 95.2% of stillbirths were identified and 4.8% were in the unclassified group. The most common causes were:

Fetal causes (64.3%), umbilical cord (14.3%), placenta (7.1%), amniotic fluid (4.8%), maternal medical conditions (2.4%). The causes of about 70% of stillbirth in Iran are unexplained, but in this study, using autopsy results and ReCoDe classification, only 4.8% of stillbirth causes remained unexplained.

**Conclusions:**

In our study, unknown cases were rare after autopsy. But considering the limitations and costs of autopsy, we need to design the guideline to specify cases who need an autopsy*.*

Fetal autopsy, placental examination and clinical information could reduce the proportion of stillbirths that remain unexplained.

**Supplementary Information:**

The online version contains supplementary material available at 10.1186/s12884-022-04822-9.

## Background

Stillbirth is a global healthcare challenge which unfortunately remains mostly neglected [1]. Even the Millennium Development Goals failed to consider many of the plans and policies addressing this issue [2]. For every 1000 total births, 18.4 stillbirths occurred worldwide in 2015 (based on WHO definition of stillbirth), most of them in low and middle income countries. Current progress towards reducing this rate is slow [3].

In many cases it is difficult to determine the certain cause of stillbirth. The cause of many cases is unexplained despite the investigations carried out, or many cases can be attributed to several factors [4]. Therefore, to find effective interventions, we need up-to-date data about the causes of stillbirth [5].

One systematic review investigated 85 reports from 50 countries, encompassing approximately 500,000 cases of stillbirth. The relevant conditions in high-income countries were unexplained in 32.1%, antepartum hemorrhage in 14.4%, placental condition in 9.3%, and congenital anomalies in 8.4% of cases, other known causes in 22.7% and other unspecified conditions in 14%. In middle-income countries, in about 43.7% of cases no specific cause is recognized found for stillbirth.

In the cross sectional study in Surinam, all hospitals in this country during 1-year (2017) reviewed and classified stillbirth causes using ICD-PM. Hypoxia occurred in 46% of cases and 41% were unclassified [6].

The situation is worse in middle-income countries; the most frequent causes were placental condition (13.7%), specific fetal/pregnancy pathologies (11.7%), antepartum hemorrhage (9.1%), other known causes (3.8%), other unspecified condition (18.7%) and unexplained (43.7%) of cases.

In low-income countries, the causes included infection in 15.8%, hypoxic peripartum death in 11.6%, antepartum hemorrhage in 9.3%, and other known causes in 8.5%, other unspecified condition in 13.8% and unexplained in 41% [7].

In a prospective observational multi-country study in sub-Saharan Africa, 1563 stillbirths were evaluated. They used healthcare providers’ opinions, an expert panel and computer-based algorithms to assign cause of death. Most common causes of stillbirth were: asphyxia (18.5–37.4%), placental disorders (8.4–15.1%), maternal hypertensive disorders (5.1–13.6%), infections (4.3–9.0%), cord problems (3.3–6.5%). 17.9–26.0% of cases remained unknown [8].

In Iran, Iranian Maternal and Neonatal Network (IMaN), has registered almost all births (live & dead), data about maternal and neonatal health electronically in and out of the hospital across the country since 2014. According to this system, the stillbirth rate during the 3 years (2014–2016) was 7.42 per 1000 births. The causes of stillbirth are not mentioned in this system [9].

Another challenge with stillbirth is the fact that more than 30 classification systems have been proposed to investigate the causes of stillbirth, and there is no agreement on a standardized international system for this purpose. Although International Classification of Disease (ICD 10, 2019 version) has a few codes related to stillbirth (including P95, Z35.2, Z37.1 and Z37.7), they are not useful for recognizing the leading causes or relevant conditions [10].

Flenady et al. evaluated different classification systems for stillbirth: Amended Aberdeen, Wigglesworth, PSANZ-PDC (Perinatal Society of Australia and New Zealand- Perinatal Death Classification), ReCoDe, Tulip and CODAC (Cause of death and associated condition). In Wigglesworth and Aberdeen many cases remained unexplained whereas CODAC and Tulip had the lowest unexplained cases. CODAC received the highest score in the ease of use score. Inter observer agreement was poor among Aberdeen and Wigglesworth. This research recommend CODAC, PSANZ-PDC, and ReCoDe for stillbirth classification [11]. So we performed ReCoDe classification because it was more likely to provide related causes of stillbirth.Gardosi et al. designed the ReCeDe system, which is a hierarchical classification system that includes primary and secondary coding. Its ultimate goal is to identify relevant conditions at the time of intrauterine death [12].

Stillbirth imposes financial burden to the family and the country’s health system and causes devastating psychological effect to the mother. Stillbirth can even affect subsequent pregnancies by influencing decisions for future pregnancies [9].

This research is designed to investigate the related causes of stillbirth through autopsy based on ReCoDe classification in a series of stillbirth cases in Iran.

## Methods

This was a retrospective study on maternal clinical records and findings of the autopsies performed on stillbirth cases. The autopsy reports of all stillbirths in Avicenna Research Institute from 2012 to 2019, were reviewed. According to the WHO and our Ministry of Health and Medical Education, stillbirth was defined as the birth of a baby with 22 or more completed weeks of gestation who died before or during labor [13].

In Iran there is not a defined protocol for referring stillbirth cases for autopsy, and the cases that are autopsied are made under the request of the doctor and the consent of the family. Moreover, the cost of an autopsy is expensive and not covered by insurance. Avicenna Research Institute is one of the advanced centers in Iran for treatment of infertility and recurrent abortion. The dead fetus is transferred to the center from different parts of the country; it is wrapped in a clean cloth, an impermeable cover, and covered with ice. Counselors ask parents questions about previous children, history of pregnancy and abortion, family history of genetic diseases, use of certain medications, and parental relationship to provide complete medical information to the diagnostic team. Then, the examination of pregnancy products (fetus, placenta and umbilical cord) is performed by an experienced pathologist. The autopsy of the fetuses was done completely, including macroscopic description, weight and measurements, internal evaluation of three parts of skull, chest, abdomen and pelvis. In addition, separate weighing of the internal organs and microscopic evaluation were done.

The complete autopsy is performed according to the guidelines mentioned in textbooks and references such as “Potter’s pathology of fetus, infant and child” and “Embryo and fetal pathology, Eind Gilbert-Barness”.

One of the limitations in this field was autopsy of dead fetuses, which were delivered more 7–10 days after death. The severe autolysis and maceration restricted the evaluation, especially of the brain.

For the diagnosis of the IUGR criteria were used in above-mentioned references, using growth curves and ratio of the brain to liver weight [14].

Organs are removed from the body, weighed and measured and samples are taken. These specimens are examined microscopically along with parts of the placenta and umbilical cord.

In some cases, at the discretion of the pathologist, genetic testing was performed. Lethality of birth defect and finding complex syndrome were possible through gross examination, autopsy results and genetic testing.

Autopsy reports are completely recorded in the patient’s file by a skilful pathologist, and one of the researchers extracted the available data by referring to the files and studying them one by one.

Data were extracted based on a checklist prepared by the researchers. The checklist contains maternal demographic information, medical history and maternal illness, pregnancy risk factors, autopsy of the fetus and placental examination. The related conditions of fetal demise were classified according to the ReCoDe system. This classification was introduced in the UK in 2005 by Jason Gardosi et al. [12]. This method have nine categories of conditions relevant to intrauterine death. These categories include fetus, umbilical cord, placenta, amniotic fluid, uterus, mother, intrapartum trauma, and unclassified conditions. The hierarchy of death-related situations is observed in this classification. This is a hierarchical classification system that includes primary and secondary coding. However, several death-related conditions can be selected [12]. Each case was classified based on ReCoDe system after thorough review of all clinical and gross or microscopic pathological findings.

In consultation with various specialists, including a gynecologist/obstetrician, an epidemiologist, pediatricians, and neonatologists who were expert in this field, we performed ReCoDe classification.

Proportion was used to describe categorical and numerical variables. Mean and SD were used to describe continuous variables. All analysis was conducted using the SPSS program version 24.0. Ethical issues of this study were approved by the medical research ethics committee in the deputy for Research Affairs of Avicenna Research Institute, Tehran, Iran. The ethics committee code is AV/FP119. At all stages of the research, the names and identities of the individuals were considered confidential. Our research have been performed in accordance with the Declaration of Helsinki. Informed consent for study participation was obtained from all parents.

## Results

In this study, stillbirth is defined as the birth of a baby with 22 or more completed weeks of gestation who died before or during labor [13]. From March 2012 to February 2019, about 220 autopsies were conducted by the Avicenna Research Institute which included miscarriage and stillbirths. All 42 cases of stillbirths were included in this study. The mean age of the mothers was 29.9 ± 4.7 years. The youngest mother was 17 years old and the oldest mother was 39 years old. Regarding the nationality, 95.2% of mothers were Iranian and 4.8% were foreign women. History of miscarriage was reported by 31.0% of women. Demographic characteristics are illustrated in Table 1.The mean age of the fathers was 34.0 ± 5.1 years (range: 25–47 years). Birth weight mean was 1134.9 ± 993.16 g and mean of gestational age was 28.51 ± 4.97 weeks.

**Table 1:** Demographic characteristics of stillbirth cases (*N* = 42)

Variables	N	%
**Child sex**		
Male	22	52.4
Female	18	42.9
Unclear	2	4.8
**Mother**^**’**^**s age**		
Less than 18 years	1	2.8
18–35	36	85.7
More than 35 years	5	11.9
**Delivery type**		
Vaginal	25	59.5
Caesarian	12	28.6
Missing data	5	11.9
**Gravidity**		
1 gravida	19	45.2
2–4 gravida	22	52.4
More than 4 gravida	1	2.4
**Parity**		
0–1	40	95.2
2–4	2	4.8
≥ 5	0	0.0
**Gestational age**		
Preterm (22–36 weeks)	37	88.1
Term (37–41 weeks)	4	9.5
Post term (≥ 42 weeks)	0	0.0
Missing data	1	2.4
**Fetus weight**		
Less than 999gr	23	54.8
1000–1499gr	6	14.3
1500–2499gr	3	7.1
2500–4000gr	4	9.5
More than 4000gr	1	2.4
Missing data	5	11.9
**Fetal stage**		
Early (22–28 weeks)	22	52.4
Late (≥ 28 weeks)	19	45.2
Missing data	1	2.4

About 70% of fetuses weighed 2,499 grams or less and 88.1% were less than 37 weeks. The average weight of the placenta was 232.08 ± 196.2 grams (in 36 of examined placenta) and the mean length of the umbilical cord was 31.74 ± 16.90 cm.

Fetus disorders were detected in 27 cases (64.3%). Congenital anomalies were related to 15 (35.7%) of stillbirths. Umbilical cord abnormalities were observed with 6 cases of (14.3%) stillbirths. One fetus distinguished with the constricting loop, and a fetus had velamentous insertion. Placental disorders were recorded in 3 cases (7.1%) of fetal demise. Placental abruption and placental insufficiency were the most common related conditions in this group. In 4.8% of cases, no relevant conditions were detected. Table 2 shows the classification of relevant conditions at death.

**Table 2:** Classification of relevant condition at death based on ReCoDe system (*N* = 42)

Group	Category	N (%)
Group A: Fetus	**Total**	**27 (64.3)**
	Lethal congenital anomaly	15 (35.7)
	Infection	1 (2.4)
	Non-immune hydrops	2(4.8)
	Iso-immunisation	1(2.4)
	Fetomaternal haemorrhage	0 (0%)
	Twin-twin transfusion	0 (0%)
	Fetal growth restriction	7 (16.7)
	Other	1 (2.4)
Group B: Umbilical cord	**Total**	**6 (14.3)**
	Prolapse	0 (0%)
	Constricting loop or knot	1 (2.4)
	Velamentous insertion	1 (2.4)
	Other	4 (9.5)
Group C: Placenta	**Total**	**3 (7.1)**
	Abruptio	1 (2.4)
	Praevia	0 (0%)
	Vasa Praevia	0 (0%)
	Placental insufficiency /infarction	1 (2.4)
	Other	1 (2.4)
Group D: Amniotic fluid	**Total**	**4 (9.6)**
	Chorioamnionitis	2 (4.8)
	Oligohydramn ios	0 (0%)
	Polyhydramnios	0 (0%)
	Other	2 (4.8)
Group E: Uterus	**Total**	**1 (2.4)**
	Rupture	0 (0%)
	Other	1 (2.4)
Group F: Mother	**Total**	**1 (2.4)**
	Diabetes	1 (2.4)
	Thyroid diseases	0 (0%)
	Essential Hypertension	0 (0%)
	Hypertensive diseases in pregnancy	0 (0%)
	Lupus/Antiphospholipid Syndrome	0 (0%)
	Cholestasis	0 (0%)
	Drug abuse	0 (0%)
	Other	0 (0%)
Group G: Intrapartum	**Total**	0 (0%)
	Asphyxia	0 (0%)
	Birth Trauma	0 (0%)
Group H: Trauma	**Total**	**0 (0%)**
	External	0 (0%)
	Iatrogenic	0 (0%)
Group I: Unclassified	**Total**	**2 (4.8)**
	No relevant condition identified	2 (4.8)
	No information available	0 (0%)
**Total**	**-**	**42 (100%)**

**Fig. 1:**
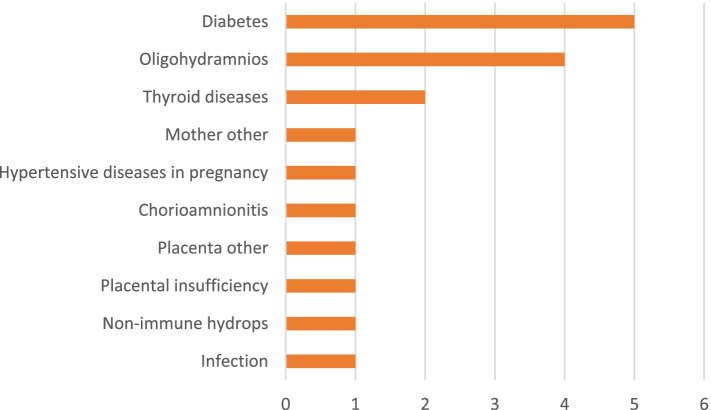
Secondary relevant conditions of stillbirth based on ReCoDe classification (*N* = 18)

Secondary relevant conditions were identified in 18 (42.9%) cases. The most frequent secondary conditions were placental disorders (50%), maternal disease (22%) amniotic fluid disorders (16.7%), and fetus disorders (11.2%). Placental insufficiency and chorionamnionitis were the most common secondary codes. The details are showed in Fig. 1.

## Discussion

In 42 cases of autopsy, based on ReCoDe classification, related causes of 95.2% of stillbirths identified and 4.8% were in the unclassified group. The most common causes were: Fetal causes (64.3%), umbilical cord (14.3%), placenta (7.1%), amniotic fluid (4.8%), and maternal medical conditions (2.4%). Among the fetal causes, the most common associated conditions were lethal congenital anomaly (35.7%), fetal growth restriction (16.7%), and non-immune hydrops (4.8%).

According to the results, this study has performed better in identifying the causes of stillbirth in Iran compared to previous studies.

We have an integrated maternity care program in Iran which includes pre-pregnancy (one care annually), prenatal care (8 cares) and postpartum (3 cares). These services are performed in health care centers by health care providers. These services are free for everyone [15].

A population-based cohort study of 2,625 stillbirth cases in West Midlands compared two classification systems: Wigglesworth and ReCoDe [12]. Wigglesworth is a simple pathophysiological classification of perinatal mortality which assigned death to one of five categories. This classification is reproducible and can be used without autopsy [16]. 66.6% of stillbirths were unexplained using the Wigglesworth classification, whereas only 15.2% of cases were unexplained using the ReCoDe classification [12]. Therefore, it seems that the use of ReCoDe system greatly reduces the unexplained.

### Congenital anomaly

The rate of congenital anomaly among stillborn varies from country to country [17]. Major anomalies are responsible for 15% to 20% fetal death [18]. A retrospective cohort study of 65,308 singleton pregnancies showed that major congenital anomalies increased the risk of stillbirth by 15-fold and even fetal growth restriction was related with a higher rate of stillbirth [19].The Ministry of Health and Medical Education has recommend screening tests including congenital anomalies and neural tube defects for all pregnant women, since 2011 [20]. This sample is not representative for all stillbirth cases, however due to the high rate of congenital anomalies in our study (35.7%), it is recommended to assess cost-effectiveness of these screening tests.

### Fetal growth restriction

Fetal growth restriction observed in about 17% in our research. It is well noted in literature that a considerable percentage of stillbirths is related to fetal growth restriction [21]. The risk of stillbirth in pregnancies with unrecognized fetal growth restriction increased over eightfold in comparison to pregnancies without fetal growth restriction [22].

Recognizing fetal growth restriction before birth is important in preventing stillbirths. Therefore, sonographic evaluation of fetal growth must be considered for all high risk patients [23].

Death at earlier gestational age (GA) is associated with congenital anomalies, intra-uterine growth restriction, and maternal medical conditions. On the other hand, at more advanced gestational ages, maternal medical conditions, obstetric disorders (such as placental abruption, placenta previa, umbilical cord prolapse, and marginal umbilical cord insertion) and unexplained causes are more frequently associated with stillbirth [24]. This was compatible with our study. About 52.4% of our stillbirth occurred at early fetal stage (22–28 weeks).

### Cord abnormalities

In this study umbilical cord abnormalities was present in about 14% of stillbirth cases. Hammad et al. evaluated 496 stillbirths and 94 (19%, 95%CI: 16–23%) of them had umbilical cord abnormality [25]. Stillbirths associated with umbilical cord abnormalities reported in 2.5 to 19% of cases in other researches [26–29]. So the results of these studies are consistent with our study.

### Placental abnormalities

To assess the causes of stillbirth, researchers in a retrospective cohort study in Italy examined 132 stillbirths from 2000 to 2004 with autopsies and placental examinations. The data were classified based on the ReCoDe system. The related cause of 79.84% identified and 20.16% were in the unclassified group. However, placental insufficiency, which occurs both in early and late stage of pregnancy, has been associated with intrauterine growth retardation. The most common secondary cause was placental abnormalities [30]. In our study placental abnormality responsible for 50% of secondary causes. Literatures confirmed that a significant percentage of stillbirth is related to placental pathology. Post-mortem examination of placenta by the skilled pathologist help to investigate the cause of stillbirth [31–33].

Accurate fetal autopsy along with placental examination and clinical information is essential for the assessment of stillbirth and can reduce unexplained cases of stillbirth [30], however lack of different resources (clinical and pathology experts, laboratory and financial resources) is the main barrier for using this approach for all cases of stillbirth. In a cohort study from 2009 to 2013 at a third level center, Miller et al. assessed 144 stillbirths step by step. Of these, 104 cases (72%) were dissected. Laboratory and clinical findings alone identified the cause of death in 35 cases. In the next step, placental pathology tests identified the probable cause of death in 61% of cases, and with the addition of autopsy, the possible causes of 74% of stillbirths were diagnosed [34].

Iranian Maternal and Neonatal Network (IMaN), registers almost all births (live & dead) electronically across the country [9]. This network recorded the relative conditions of stillbirth based on ReCoDe, but it is adjusted and it does not have enough details and it is not perfect. We still do not have the necessary resources in Iran to collect the necessary data in this field. It is noteworthy due to the fact that more than 70% of the causes of stillbirth in Iran are unknown (based on unpublished IMaN reports) [35], most of the relevant conditions are recognizable thorough review of clinical records, in addition to a simple x-ray and photography. Current system has many caveats, many of the causes would be recognizable with training and establishment of a registration system, and then we need a protocol for doing autopsy for some of the remaining unexplained cases. A review study showed that the autopsy can lead to a change in diagnosis or additional findings in 22 to 76 percent of perinatal deaths. In addition, if the confirmation of clinical findings is added, the value of autopsy can reach 100% [36].

The strength of our study was the use of autopsy report for finding related condition of stillbirths, which increased the accuracy of the results. We have some limitations such as small sample size, and missing data on some variables. In Iran, there is no defined system for which cases to be autopsied. On the other hand, we have limited resources and the centers that perform autopsies for stillbirths. Referral autopsies are performed with the consent of the family. Therefore, our sample was not representative. Another limitation was the use of the ReCoDe, which may not always distinguish between related conditions/risk factors and documented causal association.

## Conclusions

Due to fact that the cause of 70% of stillbirths in Iran is unknown, using clinical data in addition to placental examination and autopsy played an important role in identifying the related cause of stillbirth. We found related causes of 95.2% of stillbirths by using autopsy data and ReCoDe classification. In order to implement this method in this setting, it is possible to train the ReCoDe classification for the interested personnel. Institute can consider special incentives for these people.

## Supplementary Information

**Additional file 1.** Demographic-Medical-checklist.

## Data Availability

The datasets generated and/or analyzed during the current study are not publicly available due to confidentiality but are available from the corresponding author on reasonable request.

## Abbreviations

ReCoDe: Relevant Condition at Death
ICD: International Classification of Disease
PSANZ-PDC: Perinatal Society of Australia and New Zealand- Perinatal Death Classification
CODAC: Cause of death and associated condition
IMaN: Iranian Maternal and Neonatal Network
WHO: Word Health Organization
