# Supplementary material for: A retrospective autopsy study of 42 cases of stillbirth in Avicenna Research Institute
Source: BMC Pregnancy Childbirth. 2022 Jun 23;22:507. doi: 10.1186/s12884-022-04822-9 (PMC9229882; doi:10.1186/s12884-022-04822-9)
Supplement: Supplementary file 1 — Additional file 1. Demographic-Medical-checklist. [file 12884_2022_4822_MOESM1_ESM.docx]

Demographic-Medical-checklist

Demographic and medical characteristics of the mother:

| Mother's name Mother's surname |
| --- |
| Mother's ID Mother's file number |
| Mother's nationality Iranian 🞏 Non Iranian 🞏 |
| Mother's education  Mother's age: ………year Mother's Blood Group: |
| Mother's Birth Place |
| Mother's permanent residence |
| Gravidity: Parity: Number of abortions: |
| Number of children alive (from previous pregnancies): |
| Previous history of neonatal mortality: Yes 🞏 No🞏 |
| Previous history of stillbirth: Yes 🞏 No🞏 |
|  |

Delivery Risk factors:

| Delivery Risk factors: Yes 🞏 No🞏  Premature rupture of membranes: Yes 🞏 No🞏  Placental abruption: Yes 🞏 No🞏  Meconium staining: Yes 🞏 No🞏  Fetal Heart Rate Disorder Yes 🞏 No🞏  Others: …………… |
| --- |

Demographic characteristics of the father:

| Father's name: Father's surname:  Father's nationality Iranian 🞏 Non Iranian 🞏  Father's age: ………year |
| --- |

Characteristics of the stillborn:

| Birth date:  Sex: Female 🞏 Male 🞏 Ambiguous 🞏  Body weight: …………gr Height:………cm  Head circumference: ………cm  Gestational age: ………….weeks  Delivery type: Vaginal 🞏 C/S 🞏 |
| --- |

| Existence of placenta: Yes 🞏 No🞏  Placenta weight: ………..gr |
| --- |

Autopsy results:

Placental autopsy results:

Genetic test results:
